# Best practices for recruitment of adolescents for biobanking and precision health research: a retrospective analysis comparing juvenile idiopathic arthritis cases with healthy controls

**DOI:** 10.1186/s12969-021-00652-9

**Published:** 2021-12-04

**Authors:** Kimberly A. Lewis, Shelby Brooks, Ruy Carrasco, Patricia Carter, Alexandra Garcia, Jennifer Chiou, Christina Nguyen, Ambreen Rana, Sharon A. Brown, Stefano Tiziani, Nico Osier

**Affiliations:** 1 Department of Physiological Nursing, School of Nursing, University of California at San Francisco, 2 Koret Way, 94143 San Francisco, CA USA; 2 School of Nursing, The University of Texas at Austin, 1710 Red River St, 78712 Austin, TX USA; 3 Dell Children’s Medical Center of Central Texas, 4900 Mueller Blvd, 78723 Austin, TX USA; 4 Department of Physiological Nursing, University of California, San Francisco, 2 Koret Way, CA 94131 San Francisco, USA; 5 Texas A&M University, 400 Bizzell St.,, TX 77843 College Station, USA; 6 Capstone College of Nursing, University of Alabama, 650 University Blvd E, 35401 Tuscaloosa, AL USA; 7 Department of Nutritional Sciences, College of Natural Sciences, The University of Texas at Austin, Austin, TX USA; 8 Department of Neurology, Dell Medical School, The University of Texas at Austin, Austin, TX USA

**Keywords:** Healthy controls, Adolescents, Biobehavioral study, Precision health, Juvenile idiopathic arthritis, Metabolomics, Recruitment, Clinical Research, Biobanking

## Abstract

**Background:**

Precision health in adolescents relies on the successful collection of data and biospecimens from an adequately sized sample of cases and comparison group(s), often healthy controls, to answer the research question. This research report describes the recruitment strategy, enrollment rates, and approach utilized in a successful biobehavioral research study. The study was designed to examine key health indicators in adolescents (13-17 years of age) with juvenile idiopathic arthritis (JIA) compared to a control group of healthy adolescents. The purpose of this analysis is to establish best practices and identify strategies to overcome barriers to recruitment of older adolescents, an age group that tends to be underrepresented in research studies.

**Methods:**

A retrospective secondary analysis of data from a parent study about JIA with high consent rates was employed to explore factors affecting enrollment into the biobehavioral study.

**Results:**

Of the 113 subjects who were recruited to the study, 74 met the eligibility criteria and reviewed the consent form. The consented group (*n*=40) represents 54% of those who were eligible upon initial screening. The rate of project enrollment was 2.7 participants per month. The pediatric rheumatologists referred 85% of the JIA group, and the study’s principal investigator, a nurse scientist, referred 95% of the control group. Typical recruitment strategies, such as posting on social media, distributing flyers, and cold-calling potential participants from the clinic schedule were ineffective for both cases and controls. Barriers to enrollment included scheduling and fear of venipuncture. There were no demographic characteristics that significantly explained enrollment, differentiating between those who agreed to participate compared to those who refused. Successful strategies for enrollment of adolescents into this biobehavioral research study included scheduling study visits on weekends and school holidays; an informed consent and assent process that addressed adolescent fears of venipuncture; including a JIA patient on the study team; and utilizing existing relationships to maximize enrollment efforts.

**Conclusions:**

Effective recruitment and enrollment practices were relationship-specific and patient-centered. Researchers should utilize best practices to ensure that precision health for adolescents is advanced.

## Background

Juvenile idiopathic arthritis (JIA) refers to a group of autoimmune disorder sub-types affecting the synovium, or tissue around the joints, with an onset prior to age 16 years.[1, 2] All subtypes cause a release of pro-inflammatory cytokines. The inflammation leads to chronic swelling, tissue break down, and pain within one or more joints that persists greater than 6 weeks.[1, 2] JIA can affect bone growth at critical development periods and be disabling.[3].

Despite the severe impact it has on the lives of people with the disease and their caregivers, JIA is understudied.[4] The unique pathogenesis and disease course of JIA in adolescents compared to adults with rheumatoid arthritis, due in part to the influence of growth and maturation in childhood, makes studies of adults with rheumatoid arthritis an inadequate substitute for JIA research.[5, 6] Recent advances in genetics and immunology provide additional understanding and treatment guidance for individuals with the disease [7]; however, we still know very little about JIA. It is vital that research continues the search for effective treatment strategies, leading hopefully to a future cure. To achieve this recommendation, research studies first must successfully enroll participants from pediatric populations that are known to be difficult to recruit.[8] Although JIA affects all ages of children, the adolescent age group is less likely to enroll in research studies than younger children for a variety of reasons, even when access to trials is equivalent.[9, 10].

Adolescents’ attitudes toward research are generally positive, but this vulnerable population remains under-studied due to access and approval challenges, fear of research measurement techniques, scheduling complexities, and misperceptions about research.[8–14] The difficulty with recruitment increases with studies that require biospecimen collection, such as metabolomics, genomics, other -omics, and biobanking studies.[15] According to Brawner, Volpe, Stewart, & Gomes (2013), only 41% of adolescents 12-17 years of age were willing to participate in a blood draw for research purposes.[11] Without recruitment of a sufficient number of participants, the analysis may lack the power to adequately answer the research question and delay research progress; thus, delaying progress towards improving the quality of life, as well as a potential future cure, for individuals with JIA.

Very little is known about recruitment and enrollment rates for pediatric studies, including for adolescent participants.[11, 16] The few previous reports describe enrollment of pediatric patients in the clinical, school, or community settings, but none are specific to biobehavioral studies enrolling adolescent JIA cases and healthy controls and conducted in an outpatient clinical setting.[8–14, 17, 18] Schnur et al. (2019) and Paquette et al. (2018) successfully enrolled children into their biobanking studies in the emergency and critical care departments of the hospital, but these children were already receiving medical care for another reason at the time of enrollment.[13, 14].

For studies about JIA, researchers must recruit participants from a small population of adolescents with a rare disease. Without established recruitment standards, it can be challenging for researchers to plan relevant studies appropriately, allocate resources wisely, and interpret recruitment progress accurately. Inefficient recruitment methods in pediatric studies cost invaluable time and money and waste limited resources. Furthermore, recruitment of individuals from both majority and minority groups is important to ensure generalizability to more than a non-diverse sample used in a study. With the limited number of candidates, successful recruitment methods of children and adolescents are vital for finding answers to unsolved questions regarding JIA.

More evidence about recruitment is needed to advance precision health for adolescents with juvenile arthritis. The primary aim of the sub-analysis presented in this paper is to examine the screening and enrollment rates from a successful biobehavioral research study that compared adolescents 13-17 years of age with JIA to healthy adolescents (the control group). A secondary aim is to establish best practices and identify strategies to overcome barriers to participant recruitment for this age group, a population that is underrepresented in research.

## Methods

### Parent study

The parent study received human subjects’ approval from The University of Texas at Austin Institutional Review Board. The parent study aimed to describe the plasma and untargeted metabolomic signature of adolescents with JIA relative to controls, and the relationships among the metabolomic signature findings and disease characteristics, cardiovascular disease risk, biometrics (blood pressure, body mass index percentile, lipid panel, fasting blood glucose), pain, fatigue, diet, physical activity, physical function, and sleep measures. Data collection occurred between August 2018-October 2019 at the Pediatric Rheumatology outpatient clinic affiliated with the local children’s medical center in Austin, TX. This clinic serves patients from eight surrounding counties. Adolescents from both the JIA and control groups were excluded if they had a known comorbidity, had experienced any acute illness or injury within the past 7 days, or were pregnant. Participants received $50 as compensation for their time, travel, and parking costs at the clinic.

The biospecimen collection and processing procedures were standardized across all participants to minimize variation in the results. Blood was collected under fasting conditions in the morning between 800 and 1100 h via venipuncture by an experienced pediatric phlebotomist. Clean-catch urine samples were also collected and processed following the standard clinic procedure. Survey data and biometrics were collected at the clinic on the day of the study visit. Recruitment for the JIA group occurred via referral from the clinic staff; distributing flyers in the clinic, on social media, and via JIA-related community groups; and calling potential participants identified from the clinic schedule. Recruitment for the control group occurred via word of mouth, social media postings, and referrals from other participants and study team members. Interested adolescents were screened for inclusion/exclusion criteria and then scheduled for a one-hour study visit at the clinic. We encouraged participants to bring a friend to the study visit to participate with them if they felt comfortable doing so. We coordinated the study schedule to allow them to come to the clinic together for the visit whenever possible. The informed consent and assent documents were available in Spanish and English, with language translation services for study staff as needed; however, no potential participants required the Spanish consent or assent forms. Detailed screening and recruitment logs were maintained throughout the study.

### The retrospective substudy

The substudy reported here is a retrospective analysis of recruitment data from the abovementioned parent study. Data were analyzed using descriptive statistics, chi square tests, or 2-way independent samples t-tests as appropriate. Variables included: age, sex, race, ethnicity, geographic location of residence (zip code), income type (public, private, none or self-pay), parent or caregiver marital status, study group, recruitment or referral source, role of the recruiter, reason for not meeting eligibility criteria (if applicable), whether the potential participant enrolled, and reason for declining to enroll (if applicable). The reason for declining to enroll was noted based on an open-ended question asked during the recruitment process. Contemporaneous notes were taken on the recruitment and screening log then qualitative content analysis was used to code and categorize the responses. The adolescent’s sex was self-reported as male or female, as were race and ethnicity. Race was documented as White, Black, Asian or Pacific Islander, or Other with the option to select all options that apply if the adolescent identified with two or more races. Ethnicity was documented as Hispanic or Latino or not Hispanic or Latino.

## Results

**Fig. 1:**
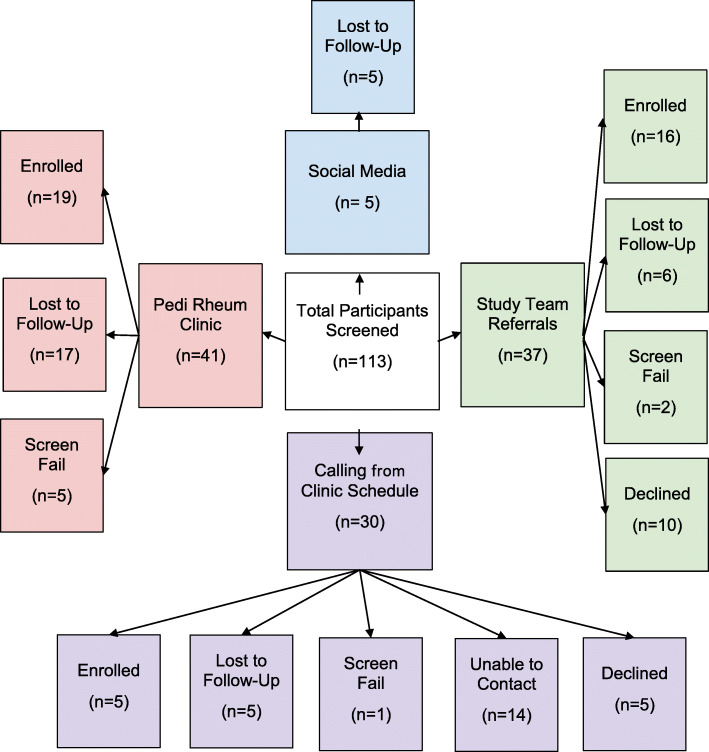
Study Recruitment and Enrollment Overview by Source

**Fig. 2:**
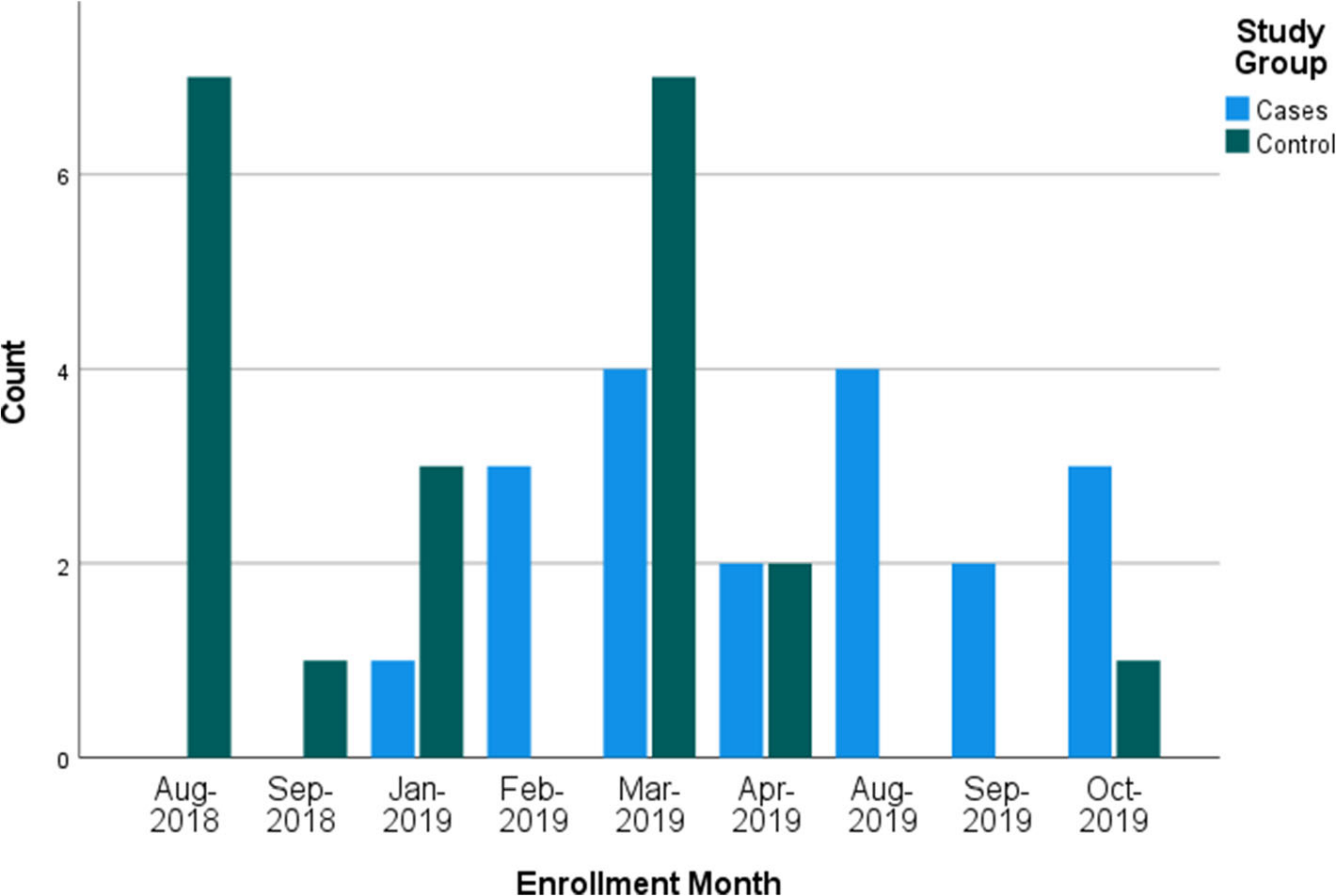
Clustered Bar Chart of Enrollment Count by Month by Study Group (Cases and Controls)

An overview of study recruitment and enrollment is depicted in Fig. 1. A total of *N*=113 potential participants were listed on the recruitment log maintained by study staff; however, 50 potential participants were either unable to be contacted initially or lost to follow-up prior to the study visit. We attempted to contact each person at least twice via phone call on separate days prior to listing them as lost-to-follow up. Of the 85 adolescents who were contacted and screened for eligibility, 47.1% of them enrolled in the study. Eight of the enrollees failed screening. Of the 74 adolescents who were both screened and eligible, the study completion rate was 54.1%. The overall rate of enrollment for the entire study period was 2.67 participants per month. Figure 2 provides an overview of study enrollment by month.

### Recruitment source

Table 1 details enrollment by recruitment source. The highest number of enrollees were identified through pediatric rheumatology physician referral (17/20 adolescents with JIA, or 85%), or through personal and professional networks of the principal investigator (19/20 controls, or 95%). The clinics participating in this study did not have physicians’ assistants or nurse practitioners, so it is unknown how that recruitment source may have contributed to successful enrollment. Social media posts (i.e., Twitter and Facebook) increased the number of referrals, but no cases or controls were enrolled from this recruitment source. Cold-calling potential participants linked to the clinic schedule was the least effective method other than social media. A total of 30 potential participants with JIA were identified through the clinic schedule and called by study volunteers, but only 5 from this recruitment source enrolled in the study (16.7% of those screened, 12.5% of the total sample). The Hispanic and Latino adolescents with JIA enrolled exclusively when recruited by the Hispanic pediatric rheumatologist than any other recruiter. Hispanic or Latino controls who enrolled were recruited equally by the principal investigator (*n*=4) and the Hispanic pediatric rheumatologist (*n*=4). Male JIA cases were more likely to enroll when recruited by the pediatric rheumatologist (57% success). Male controls were more likely to enroll when recruited by the principal investigator (50% success).

**Table 1:** Recruitment Summary by Source and Reason Code for Cases and Controls

Source	Code	Controls	Cases	Total
**Pedi Rheum Clinic**	**Total from Source**	**9**	**32**	**41**
	Enrolled	6	13	19
	Lost to Follow-up	3	14	17
	Screen Fail (4 age, 1 comorbidity)	-	5	5
**Calling from Clinic Schedule**	**Total from Source**	**-**	**30**	**30**
	Enrolled	-	5	5
	Lost to F/U	-	5	5
	Screen Fail (comorbidity)	-	1	1
	Declined (2 unfamiliar with study team, 3 unknown or prefer not to say)	-	5	5
	Unable to Contact	-	14	14
**Study Team Referrals – Principal Investigator**	**Total from Source**	**28**	**-**	**28**
	Enrolled	14	**-**	14
	Lost to F/U	4	**-**	4
	Screen Fail (age)	1	**-**	1
	Declined (1 scheduling, 3 afraid of needles, 1 afraid of doctors, 4 unknown)	9	**-**	9
**Study Team Referrals – Study Recruitment Team**	**Total from Source**	**4**	**5**	**9**
	Enrolled	1	1	2
	Lost to F/U	1	4	5
	Screen Fail (recent surgery)	1	**-**	1
	Declined (fear of blood draw)	1	**-**	1
**Social Media (Facebook and Twitter)**	**Total from Source**	**5**	**-**	**5**
	Lost to Follow Up	5	**-**	5
***Total Referrals***		***46***	***67***	***113***

### Enrollment barriers

There were two barriers to enrollment: scheduling and fear of the venipuncture. Scheduling the morning fasting blood draw was the main barrier noted until we offered appointments on weekends and school holidays. In addition, potential participants stated that the blood draw was a source of their anxiety about the study. At least a third of the control group participants reported that they had never had a blood draw prior to our study. Some stated that they imagined that very large quantities of blood would be drawn. Adolescents with JIA, who frequently must undergo blood draws for disease monitoring and intravenous catheter insertions for drug infusions, were concerned about having additional needle sticks. The strategies employed to address these concerns included: addressing needlestick misconceptions (such as quantifying the total amount of blood drawn in familiar terms) during the consenting process, hiring an experienced pediatric phlebotomist, and combining the study visit with a JIA infusion appointment or routine blood draw whenever possible to minimize needle sticks.

### Characteristics of enrolled participants

**Fig. 3:**
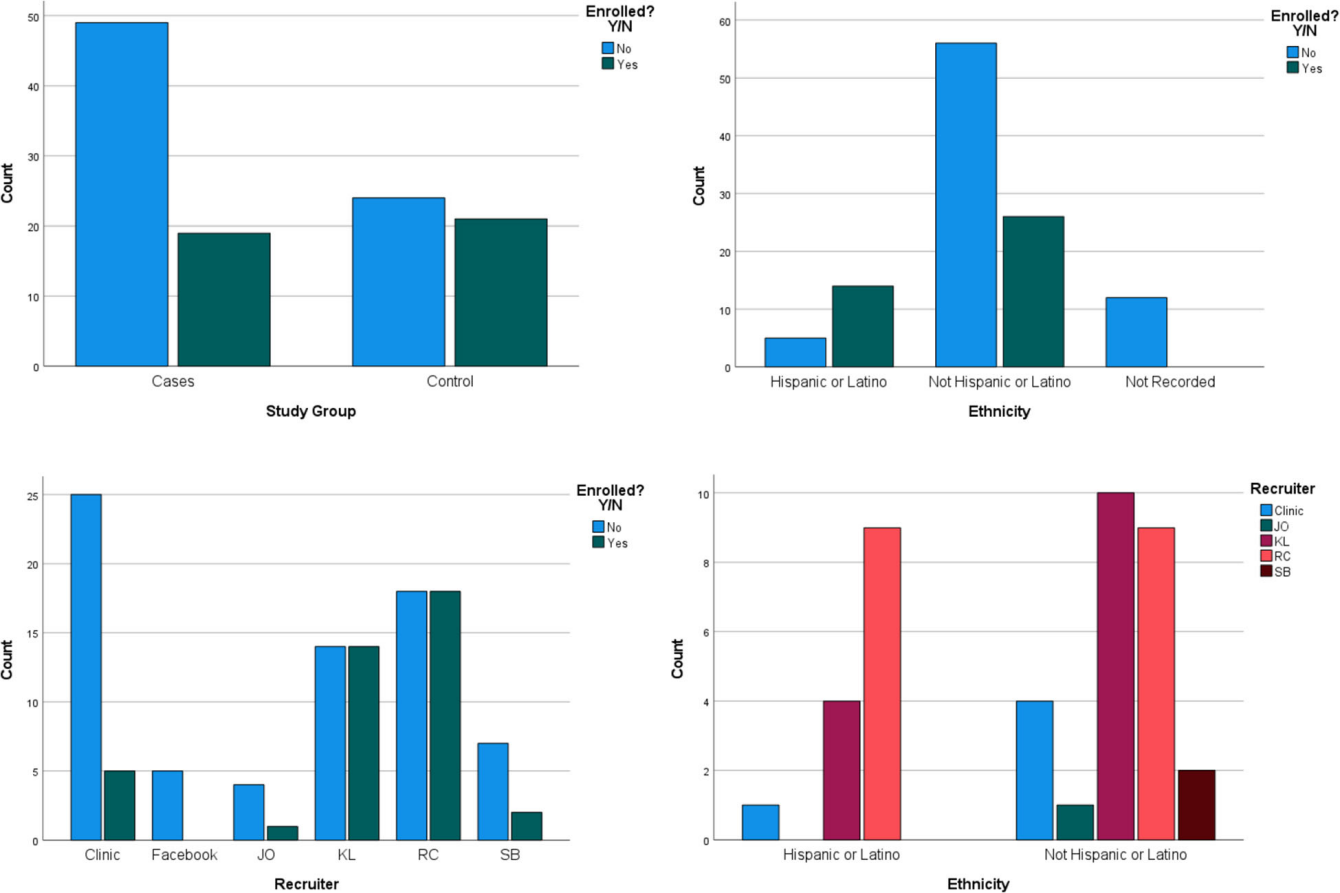
Comparison of Characteristics of the Referrals Enrolled and Not Enrolled

Characteristics of potential participants contacted, screened, and enrolled are described in Tables 2 and 3; Fig. 3. There was no significant difference in the mean age of those who enrolled (14.8 ± 1.5 years) and those who declined (14.7 ± 1.8 years, *p*=0.96). The ratio of the adolescents who were screened to those enrolled differed significantly between JIA cases (2.5:1) vs. controls (1:1); males (2.5:1) vs. females (1:1); and ethnicity (Hispanic/Latinos 1:2.5 vs. non-Hispanic white 1:1). Nearly two-thirds of all Hispanic/Latino patients enrolled were referred by their pediatric rheumatologist. The remaining 29% was referred from the study principal investigator, and 7% from the clinic schedule. As detailed in Tables 3 and 26% of participants in the JIA group utilized public insurance compared to 10% of the control group. Most participants in both JIA and control groups lived in two-parent households. Enrolled participants traveled to the clinic from 7 of the surrounding counties, ranging from approximately 5-161 miles, with an approximate mean distance of 34 miles traveled to the clinic. Enrolled participants in the JIA group traveled an approximate mean distance of 34.3 miles compared with 27.7 miles for the control group (*p*=0.72).

**Table 2:** Comparison of Characteristics of Participants Enrolled and Not Enrolled

Characteristic	Enrolled (n, %)	Not Enrolled (n, %)	χ2 or *t* (p value)
**Ethnicity** Hispanic or Latino Not Hispanic or Latino Not Recorded	14 (35%) 26 (65%) -	5 (7%) 56 (67%) 12 (16%)	**χ2** =19.24 (*p*<0.001)
**Sex** Male Female	12 28	29 31	**χ2** =11.56 (*p*=0.003)
**Study Group** Cases Controls	19 21	49 24	**χ2** =4.15 (*p*=0.042)
**Age (Mean)**	14.8 ± 1.5	14.7 ± 1.8	*t*=0.052 (*p*=0.958)

**Table 3:** Insurance Type and Parent Marital Status of Enrolled Participants by Study Group (Juvenile Idiopathic Arthritis or Control)

	JIA Group (*n*=19)		Control Group (*n*=20)			
**Descriptor**	**n**	**%**	**n**	**%**	**χ**^**2**^	***p*value**
Insurance Type						
Public	5	26%	2	10%	3.07	0.22
Private	13	68%	18	90%		
None or Self Pay	1	5%	0	-		
Parent Marital Status						
Married	17	89%	16	80%	1.21	0.55
Single	0	-	1	5%		
Divorced	2	11%	3	15%		
Separated	0	-	0	-		

JIA=Juvenile Idiopathic Arthritis; Public Insurance=Medicare, Medicaid, other Government-Provided Healthcare Insurance

## Discussion

This paper is the first to describe the successful recruitment and enrollment of older adolescents with JIA and healthy controls into a biobehavioral metabolomics study with a morning, fasting biospecimen collection. There was no difference in the mean age of the referrals who enrolled compared to referrals who declined, but there were other characteristics (self-reported sex, ethnicity, and study group) that contributed to enrollment. Our findings affirm that recruitment strategies may be more effective if customized by study group (i.e., JIA cases or controls) and the target of the recruitment (i.e., parents/caregivers or adolescent). This study also highlighted multiple mechanisms for enhancing recruitment of adolescents to studies on JIA including diversity within the study team, personal connection to recruiters, timing of enrollment, and utilization of volunteers.

Enrollment as a percentage of total screened in this study was high compared to other published studies which reported enrollment rates of 35–62%.[18] This high recruitment rate was especially true for the control group of healthy adolescents in our study. As reported by Kong et al. (2013), usually clinic-based adolescent referrals (i.e., those in the JIA group) and their parents are more likely to participate in clinical research.[19] However, we had no difficulty enrolling control group adolescents into this biobehavioral study.

Consideration for the desired audience (either the parents or the adolescents themselves) should be made while planning recruitment strategies. For example, the social media sites (Facebook, Twitter) used in this study were ineffective methods of recruitment. These sites primarily reach the parents or caregivers, and it may be that the perceived risk for enrolling their adolescents in a biobehavioral research study is too great a barrier to overcome via social media. However, newer social media sites with high adolescent engagement like TikTok, Instagram, or Snapchat may be more effective ways to reach potential adolescent participants.[20].

Similar to research studies about adult participants, potential participants who self-identified as female were more likely to enroll than males. In this JIA study, controls were more likely to enroll than JIA cases; and adolescents who self-identified as Hispanic or Latino more likely than not Hispanic or Latino adolescents. Older adolescents are less likely than other pediatric age groups to enroll in clinical trials.[9] Evidence is mixed about the proportion of females and males that enroll in clinical trials.[21] It may be that enrollment is diagnosis specific. For example, in cancer research trials enrolling adolescent participants, the higher proportion of enrolled males was also in the presence of a greater number of eligible males.[21] In JIA, females are affected twice as often as boys so there was a larger pool of potential females with JIA to enroll.[4] Another potential factor in the success of the present study is that we encouraged all our participants to bring a friend to the study visit with them if they felt comfortable doing so. This was particularly effective for the females in our sample.

Our findings underscore the importance of representation and diversity in the study team to enroll diverse samples. Hispanic or Latino adolescents are grossly underrepresented in clinical research trials, particularly those that involve biospecimen collection. [15, 22, 23] As a result, we consider it to be a great success that the proportion of Hispanic or Latino participants who enrolled in our study after referral compared to those who declined was so high. As described by Garcia et al. (2017), a personal connection and direct contact with the research team are important to enroll Hispanic or Latino participants.[24] Our pediatric rheumatologist had established relationships with his patients. He was also fluent in Spanish and was able to communicate with potential participants without a translator. All these strategies are consistent with Hispanic/Latino values and contribute to successful enrollment.[25].

When planning for study timelines, we found that the rate of enrollment for this study coincided with the academic calendar. During the school year, enrollment slowed until we began offering study appointments on weekends and school holidays. Spring break (March) and the end of summer break (August) were the most effective, especially for the control group participants, but we were also successful at enrolling during three-day weekends and winter holidays throughout the year. Findings suggest that targeted summer recruitment efforts should aim for the end of summer break, coinciding with return to school activities, instead of the beginning or middle when participants may be traveling, participating in summer camps, or fatigued from school obligations. Scheduling during clinic off-hours (i.e., weekends and holidays) allowed us to utilize the clinic and lab spaces without competing with the normal clinic schedule. These factors contributed to our enrollment rate, which was high relative to other adolescent studies.[14].

Utilizing volunteers helped us to be more successful with recruitment, especially for offering weekend and holiday scheduling while also keeping costs low. Graduate students from the university and trained research volunteers from the hospital system’s formal research volunteer program assisted with laboratory processing, data entry, and recruitment. Including a patient stakeholder on the research team is a recommendation supported by many experienced professionals, including the Arthritis Foundation, Childhood Arthritis and Rheumatology Research Alliance, and practicing clinicians. [25]

Another option to increase enrollment is to offer in-home study visits. Enrolled cases and controls in our study traveled equivalent distances to the clinic on average, and home visits were not used. However, incorporating home visits may be particularly useful for adolescents with rare diseases like JIA, because a single clinic may serve a broad surrounding geographic area.[15] Transportation to the clinic from remote areas can be a barrier to enrollment, because not only may adolescents need to miss school, but parents may also need to miss a day off work to travel.[16] This strategy may also be particularly useful for recruiting a sample that includes people who are traditionally underrepresented in research.[15] However, in-home study visits can be costly in terms of staff time and travel costs, so these issues must be considered.

Although this study makes important contributions to the literature about recruitment and enrollment of older adolescents in research, there are limitations. The study was conducted at a single site with a small sample size, limiting the generalizability to the entire population of JIA and control group samples. These findings should be confirmed in a larger sample and in other settings. We were unable to follow up with those adolescents who declined to enroll, thus limiting our ability to understand with greater depth the factors that would promote or limit enrollment. Future studies should investigate additional factors and participant and parent characteristics, such as ethnic groups beyond Hispanic or Latino, socioeconomic status, and health literacy of the adolescents and their parents. Additional formal investigation of the effectiveness of each of the interventions, perhaps via focus groups or surveys distributed to parents and adolescents, would provide additional insight into the most effective strategies overall and by subgroup.

## Conclusions

The goal of this substudy inquiry is to promote the enrollment of adolescent samples in biobehavioral clinical research studies that are representative of the diverse United States population. Such evidence will support precision health initiatives and the care of patients with rare diseases like JIA. Our findings underscore the importance of active recruitment strategies by the principal investigator, pediatric rheumatologist, and the other clinic and research team members with existing ties to the community being studied. Building a diverse, representative research team that reflects the community being studied may lead to enrollment success. For adolescent precision health to advance, research teams must employ creative recruitment and scheduling strategies to meet the needs of the population.

## Data Availability

All data generated or analysed during this study are included in this published article.

## Abbreviations

JIA: Juvenile Idiopathic Arthritis
